# Oral Efficacy of Apigenin against Cutaneous Leishmaniasis: Involvement of Reactive Oxygen Species and Autophagy as a Mechanism of Action

**DOI:** 10.1371/journal.pntd.0004442

**Published:** 2016-02-10

**Authors:** Fernanda Fonseca-Silva, Job D. F. Inacio, Marilene M. Canto-Cavalheiro, Rubem F. S. Menna-Barreto, Elmo E. Almeida-Amaral

**Affiliations:** 1 Laboratório de Bioquímica de Tripanosomatídeos, Instituto Oswaldo Cruz, Fundação Oswaldo Cruz, Manguinhos, Rio de Janeiro, Brazil; 2 Laboratório de Biologia Celular, Instituto Oswaldo Cruz, Fundação Oswaldo Cruz, Manguinhos, Rio de Janeiro, Brazil; The Ohio State University, UNITED STATES

## Abstract

**Background:**

The treatment for leishmaniasis is currently based on pentavalent antimonials and amphotericin B; however, these drugs result in numerous adverse side effects. The lack of affordable therapy has necessitated the urgent development of new drugs that are efficacious, safe, and more accessible to patients. Natural products are a major source for the discovery of new and selective molecules for neglected diseases. In this paper, we evaluated the effect of apigenin on *Leishmania amazonensis in vitro* and *in vivo* and described the mechanism of action against intracellular amastigotes of *L*. *amazonensis*.

**Methodology/Principal Finding:**

Apigenin reduced the infection index in a dose-dependent manner, with IC_50_ values of 4.3 μM and a selectivity index of 18.2. Apigenin induced ROS production in the *L*. *amazonensis*-infected macrophage, and the effects were reversed by NAC and GSH. Additionally, apigenin induced an increase in the number of macrophages autophagosomes after the infection, surrounding the parasitophorous vacuole, suggestive of the involvement of host autophagy probably due to ROS generation induced by apigenin. Furthermore, apigenin treatment was also effective *in vivo*, demonstrating oral bioavailability and reduced parasitic loads without altering serological toxicity markers.

**Conclusions/Significance:**

In conclusion, our study suggests that apigenin exhibits leishmanicidal effects against *L*. *amazonensis*-infected macrophages. ROS production, as part of the mechanism of action, could occur through the increase in host autophagy and thereby promoting parasite death. Furthermore, our data suggest that apigenin is effective in the treatment of *L*. *amazonensis*-infected BALB/c mice by oral administration, without altering serological toxicity markers. The selective *in vitro* activity of apigenin, together with excellent theoretical predictions of oral availability, clear decreases in parasite load and lesion size, and no observed compromises to the overall health of the infected mice encourage us to supports further studies of apigenin as a candidate for the chemotherapeutic treatment of leishmaniasis.

## Author Summary

Leishmaniasis is an important neglected disease caused by protozoa of the genus *Leishmania* and affects more than 12 million people worldwide. Pentavalent antimonials and amphotericin B have been used for decades to treat leishmaniasis; however, these drugs result in numerous adverse side effects, have variable efficacy and are subject to parasite resistance. The lack of suitable therapy necessitates the development of novel antileishmanial compounds. In this study, we investigated the antileishmanial activity of apigenin *in vitro* and *in vivo* and described the mechanism of action against intracellular amastigotes of *Leishmania amazonensis*. Apigenin reduced the infection index in a dose-dependent manner and increased reactive oxygen species (ROS) generation. Additionally, apigenin induced an increase in the number of macrophages autophagosomes after the infection, surrounding the parasitophorous vacuole, suggestive of the involvement of host autophagy probably due to ROS generation induced by apigenin. Furthermore, treatment with apigenin was also effective *in vivo*, showing oral bioavailability and significantly reducing lesion sizes and parasite burden without altering serological toxicity markers.

## Introduction

Leishmaniasis is a parasitic disease endemic in 98 countries, affecting more than 12 million people worldwide. Cutaneous leishmaniasis has an incidence of approximately 1.2 million cases per year [1]. *Leishmania amazonensis* is the etiological agent of cutaneous or diffuse cutaneous lesions. Originally described in the Amazon region, *L*. *amazonensis* occurs in many parts of Brazil [2]. Pentavalent antimonials, the first-line compounds, and amphotericin B, second-line drugs, have been used for decades to treat leishmaniasis, saving thousands of lives. However, these treatments require intra-muscular administration and long periods of internalization have several side effects and contribute to parasite resistance, reducing the efficacy of treatment [3,4]. The lack of affordable therapy has necessitated the urgent development of new drugs that are efficacious, safe, and more accessible to patients.

Natural products are a major source for the discovery of new and selective molecules for neglected diseases [5,6]. Compounds isolated from plants, including some flavonoids, have been reported to possess significant antiprotozoal properties [7–13]. Apigenin (5,7,4'-trihydroxyflavone) is a natural flavone that is abundantly found in fruits and vegetables such as parsley, lemons and berries. It has been recognized as a bioactive flavonoid with a wide range of reported biological effects, including antioxidant, cancer chemopreventive, antihypertensive, anti-inflammatory, antimicrobial and antiprotozoal activities.[14–20] It has been shown that apigenin induces mitochondrial damage and the production of reactive oxygen species (ROS) [16–20]. However, the precise molecular mechanisms underlying its antiprotozoal activity remain unknown.

In this study, we describe the possible mechanism of action for apigenin, demonstrating that antileishmanial activity *in vitro* against intracellular amastigotes of *L*. *amazonensis* and *in vivo*, using a murine-model of cutaneous leishmaniasis. Apigenin reduced the infection index in a concentration-dependent manner. Additionally, apigenin demonstrated to be non-cytotoxic to murine macrophages at a potent leishmanicidal concentration, with activity that was determined to be ROS-dependent. *Leishmania*-infected macrophages treated with apigenin exhibited an increase in double-membrane vesicles and myelin-like membrane inclusions, characteristic of autophagosomes. Apigenin treatment was also effective in a murine model of *L*. *amazonensis* infection, demonstrating oral bioavailability, as it decreased parasitic load without altering serological toxicology markers.

## Materials and Methods

### Test Compound and Reagents

Apigenin (≥97% purity; lot 081M1457V), Schneider's *Drosophila* medium, fetal calf serum, RPMI-1640 medium, penicillin, streptomycin, JC-1 (5,5′,6,6′-tetrachloro-1,1′,3,3′-tetraethyl-imidacarbocyanine iodide), NAC (*N*-acetyl-L-cysteine), GSH (reduced glutathione), glutaraldehyde, sodium cacodylate, osmium tetroxide, potassium ferricyanide, uranyl acetate and FCCP [carbonyl cyanide 4-(trifluoromethoxy)phenylhydrazone] were obtained from Sigma-Aldrich (St. Louis, MO, USA). H_2_DCFDA (2',7'-dichlorodihydrofluorescein diacetate) was obtained from Invitrogen Molecular Probes (Leiden, The Netherlands). All other reagents were purchased from Merck (São Paulo, Brazil). Deionized distilled water obtained using a Milli-Q system (Millipore Corp., Bedford, MA, USA) was used to prepare all solutions. Endotoxin-free sterile disposable supplies were used in all experiments. Apigenin was prepared in dimethylsulfoxide (DMSO) and diluted in culture medium such that the solvent concentration did not exceed 0.2% (v/v) in the final solution. In the control samples (absence of apigenin), a similar volume of vehicle (DMSO 0.2% v/v) was added to the cells.

### Parasites

The MHOM/BR/75/LTB0016 strain of *L*. *amazonensis* was used throughout this study. This strain was isolated from a human case of cutaneous leishmaniasis in Brazil. Promastigotes were cultivated at 26°C in Schneider medium (pH 7.2) supplemented with 100 U/mL penicillin, 100 μg/mL streptomycin and 10% (v/v) heat-inactivated fetal calf serum.

### Leishmania-Macrophage Interaction Assay

*L*. *amazonensis* promastigotes were washed with phosphate-buffered saline (PBS), counted using a Neubauer chamber and added to peritoneal macrophages at a multiplicity of infection (MOI) of 3.0. The macrophages were collected from Swiss mice (6–8 weeks old), plated in Roswell Park Memorial Institute (RPMI) medium at 2 × 10^6^ cells/mL (0.4 mL/well) in Lab-Tek eight-chamber slides and then incubated for 3 h at 37°C in an atmosphere of 5% CO_2_. The free parasites were removed by successive washes with RPMI medium. *L*. *amazonensis*-infected macrophages were then incubated in the absence or in the presence of apigenin (3 μM, 6 μM and 12 μM) for 72 h. The percentage of infected macrophages was determined using light microscopy; at least 300 cells on each coverslip were counted randomly in duplicate. The results were expressed as the infection index (% of infected macrophages × number of amastigotes/total number of macrophages). The IC_50_ value was determined by logarithmic regression analysis using GraphPad Prism 6. In the control samples (absence of apigenin), a similar volume of vehicle (DMSO 0.2% v/v) was added to the cells. The experiments were performed thrice.

### Viability Assay

Peritoneal macrophages (2 × 10^6^ cells/mL) were allowed to adhere to 96-well tissue culture plates for 1 h at 37°C in an atmosphere of 5% CO_2_. Non-adherent cells were removed by washing with RPMI-1640 medium. Then, the adherent macrophages were incubated with the indicated concentrations of apigenin (3 to 96 μM) for 72 h. The medium was then discarded, and the macrophages were washed with RPMI-1640, after which time they were incubated with Alamar blue (10% v/v) for 12 h at 37°C in an atmosphere of 5% CO_2_. The absorbance was measured at 570 nm using a spectrophotometer, and the IC_50_ value was determined by logarithmic regression analysis using GraphPad Prism 6. The selectivity index was determined as macrophage IC_50_/intracellular amastigote IC_50_. Untreated peritoneal macrophages were lysed by the addition of 0.1% Triton X-100 as a positive control.

### Determination of ΔΨ_m_

Peritoneal macrophages (2 × 10^6^ cells/mL) were allowed to adhere to black 96-well tissue culture plates for 1 h at 37°C in an atmosphere of 5% CO_2_. Non-adherent cells were removed by washing with RPMI-1640 medium. Next, the adherent macrophages were incubated with the indicated concentrations of apigenin (3 μM, 6 μM and 12 μM) for 72 h. Cells were harvested and resuspended in Hank's Balanced Salt Solution (HBSS) and incubated with JC-1 (10 μg/mL) for 30 min at 37°C in an atmosphere of 5% CO_2_. After washing twice with HBSS, fluorescence was measured spectrofluorometrically at 530 nm and 590 nm using an excitation wavelength of 480 nm. The ratio of values obtained at 590 nm and 530 nm was plotted as the relative ΔΨ_m_. The mitochondrial uncoupling agent carbonyl cyanide *p*-(trifluoromethoxy)phenylhydrazone (FCCP; 200 μM) was used as a positive control.

### Measurement of ROS Levels

Intracellular ROS levels in uninfected macrophages and in *L*. *amazonensis*-infected macrophages that were treated with apigenin or untreated were measured using the cell-permeable dye H_2_DCFDA. *L*. *amazonensis* promastigotes were added to the peritoneal macrophages at an MOI of 3.0. The cells were then plated in black 96-well tissue culture plates in RPMI-1640 medium at a density of 2 × 10^6^ macrophages/mL and incubated for 3 h at 37°C in an atmosphere of 5% CO_2_. For the uninfected macrophages, peritoneal macrophages were plated in black 96-well tissue culture plates at a density of 2 × 10^6^ macrophages/mL and incubated for 3 h at 37°C in the presence of 5% CO_2_. Uninfected macrophages and *L*. *amazonensis*-infected macrophages were incubated in the absence or presence of apigenin (3 μM, 6 μM and 12 μM) for 72 h. The medium was then discarded, the macrophages were washed with HBSS, and the cells were incubated with H_2_DCFDA (20 μM) for 30 min at 37°C. Fluorescence was measured spectrofluorometrically using an excitation wavelength of 507 nm and an emission wavelength of 530 nm. For all measurements, basal fluorescence was subtracted.

### Transmission Electron Microscopy Analysis of *L. amazonensis*-Infected Macrophages

*L*. *amazonensis* promastigotes were added to the peritoneal macrophages at an MOI of 3.0. Next, *L*. *amazonensis*-infected macrophages were incubated in the absence or in the presence of apigenin (12 μM) for 72 h. After washing with PBS, the infected macrophages were fixed in 2.5% glutaraldehyde in 0.1 M sodium cacodylate buffer (pH 7.2) at room temperature for 40 min and post-fixed in a solution of 1% osmium tetroxide, 0.8% potassium ferricyanide and 2.5 mM CaCl_2_ for 20 min. The cells were dehydrated in an acetone series and embedded in PolyBed 812 resin.[21] Ultrathin sections were stained with uranyl acetate and lead citrate and examined using a JEOL JEM1011 transmission electron microscope (Tokyo, Japan) in the Plataforma de Microscopia Eletrônica, IOC, FIOCRUZ.

### *In Vivo* Infection in the Murine Model

BALB/c mice (5/group) were maintained under specific pathogen-free conditions and inoculated with stationary-phase *L*. *amazonensis* promastigotes (2 x 10^6^ cells in 10 μl of PBS) intradermally in the right ear using a 27.5-gauge needle. The method of treatment was similar to previously described methods [9] and was initiated seven days following infection. Apigenin (1 mg/kg and 2 mg/kg) was diluted in DMSO (1% v/v), incorporated in an oral suspension and administered orally through an orogastric tube once daily seven times per week until the end of the experiment (day 45), when the animals were euthanized. The control group was treated orally with an oral suspension with DMSO (1% v/v) in the absence of apigenin (vehicle of apigenin). The positive control was treated with intraperitoneal injections of meglumine antimoniate (pentavalent antimonial; 100 mg/kg/day) once daily seven times per week until the end of the experiment (day 45). The lesion sizes were measured twice per week using a dial caliper.

### Parasite Load Quantification

The parasite load was determined 45 days post-infection using a quantitative limiting dilution assay as described previously [9,13]. The infected ears were excised, weighed and minced in Schneider's medium with 20% fetal calf serum. The resulting cell suspension was serially diluted. The number of viable parasites in each ear was estimated from the highest dilution that promoted promastigote growth after seven days of incubation at 26°C.

### Toxicology

Serum levels of toxicological markers (aspartate aminotransferase (AST), alanine aminotransferase (ALT), creatinine (CREA), urea, total protein (TP), globulin (GLO), albumin (ALB) and creatine kinase (CK) in the infected BALB/c mice treated as described above were measured by the Program of Technological Development in Tools for Health-PDTIS-FIOCRUZ.

### Ethics Statement

This study was performed in strict accordance with the recommendations of the Guide for the Care and Use of Laboratory Animals of the Brazilian National Council of Animal Experimentation (COBEA). The protocol was approved by the Committee on the Ethics of Animal Experiments of the Fundação Oswaldo Cruz (CEUA-FIOCRUZ, License Number: LW-7/10).

### Statistical Analysis

All experiments were performed in three independent trials. The data were analyzed using Student’s t-test or analysis of variance (ANOVA) followed by Bonferroni's post-test in GraphPad Prism 6 (GraphPad Software, La Jolla, CA, USA). The results were considered significant when *p*≤ 0.05. The data are expressed as the mean ± standard error.

## Results and Discussion

**Fig 1:**
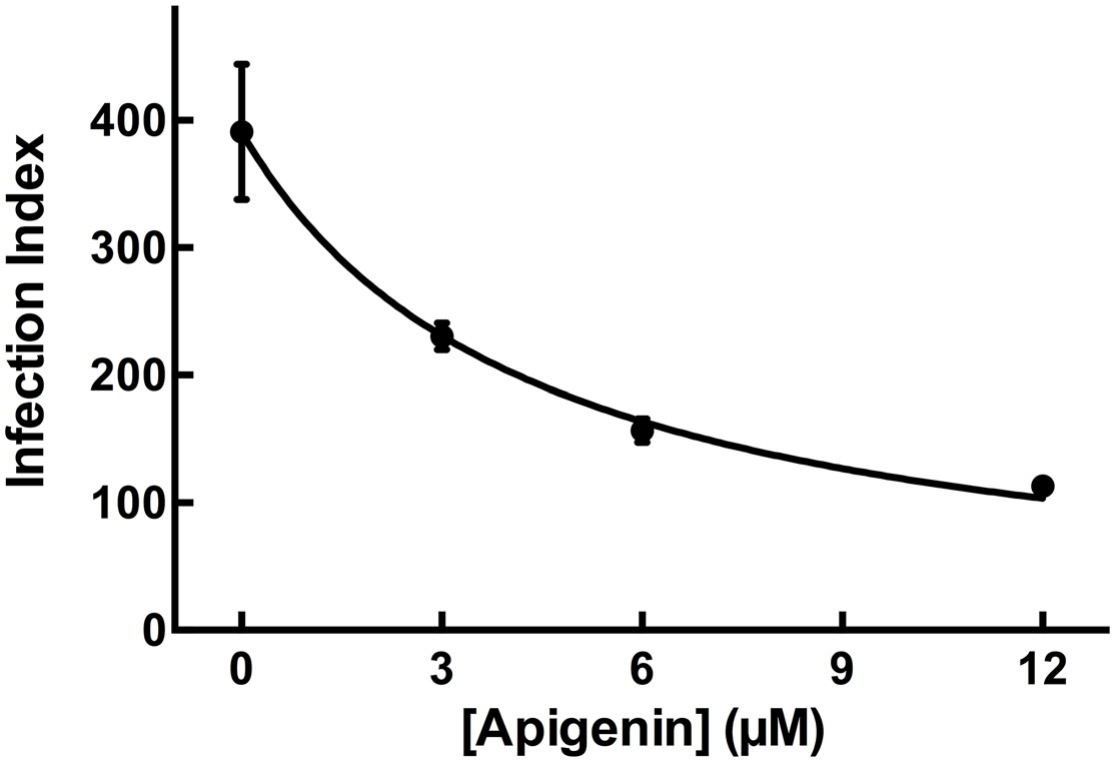
Effect of apigenin on *L*. *amazonensis*-infected macrophages. Macrophages were infected with *L*. *amazonensis* promastigotes for 3 h at 37°C and incubated in the absence or presence of apigenin (3 μM, 6 μM and 12 μM) for 72 h. The infection index was determined using light microscopy; at least 300 macrophages were counted on each coverslip in duplicate. The values shown represent the mean ± standard error of three independent experiments. In the control samples (absence of apigenin), a similar volume of vehicle (0.2% DMSO) was added to the cells. n = 3.

The activity of apigenin against the promastigote form of *L*. *amazonensis* has been described [17]. To determine the effects of apigenin on the interaction of *L*. *amazonensis* with macrophages after parasite invasion, untreated promastigotes were allowed to interact with macrophages for 3 h. *Leishmania*-infected macrophages were then incubated in the absence or presence of apigenin (3–12 μM) for 72 h (Fig 1). Apigenin reduced the infection index in a dose-dependent manner (*p <* 0.001) with an IC_50_ value of 4.3 μM, and inhibited the growth of *L*. *amazonensis* by 71% after 72 h at the highest dose tested (12 μM).

An evaluation of the cytotoxic effect of apigenin in murine macrophages revealed a lack of toxicity and maintenance of the mitochondrial membrane potential (S1 Fig). The IC_50_ value of apigenin against murine macrophages was 78.7 μM, which corresponds to a selectivity index of 18.2. The biological efficacy of a drug is not attributed to cytotoxicity when the selectivity index is greater than or equal to 10 [22,23]. These results demonstrate the antileishmanial activity of apigenin against *L*. *amazonensis* amastigotes.

ROS are produced as a response to pathogen infection of macrophages and result in the destruction of cellular and macromolecular components. ROS can also be generated in response to the administration of some drugs; this mechanism is the basis of various antiprotozoal medications used to combat parasites in infected cells [24]. Although apigenin is known to exhibit antioxidant properties, some studies have demonstrated pro-oxidant activities, resulting in cytotoxicity in some cancer cells [16,19,20].

**Fig 2:**
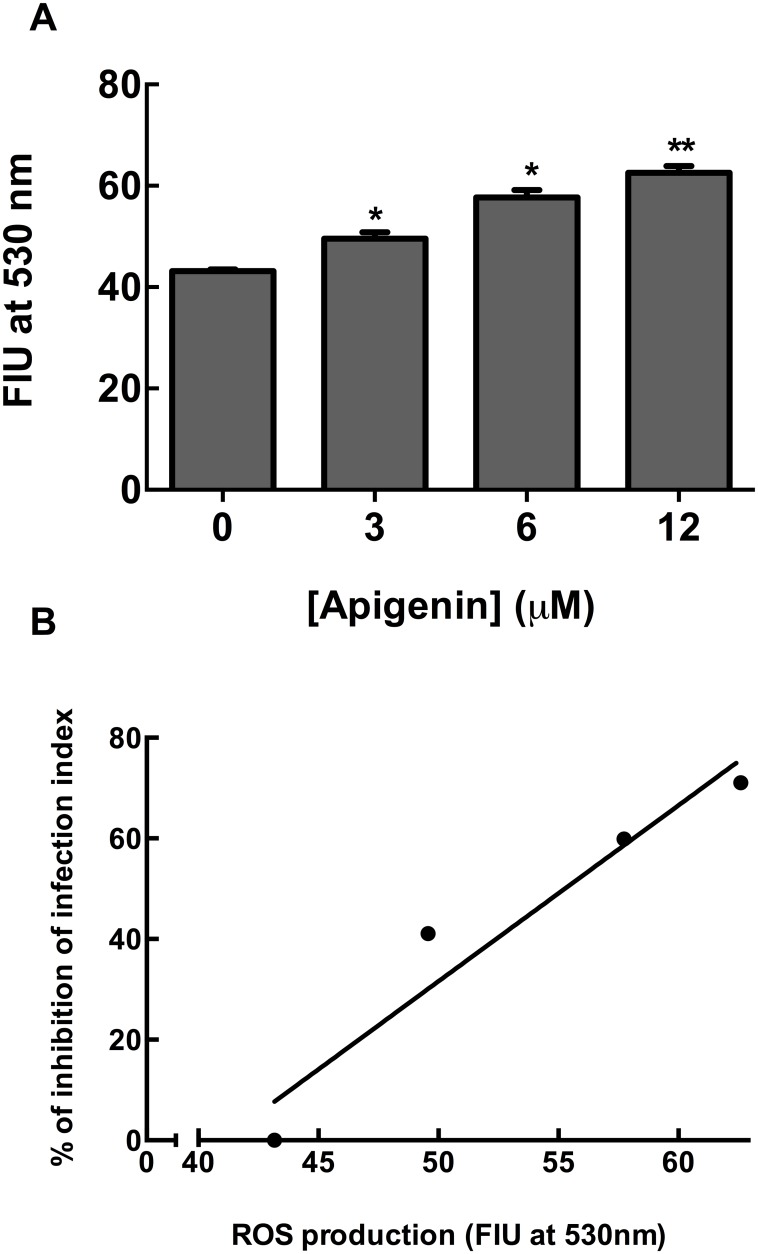
Apigenin-induced ROS generation. *L*. *amazonensis*-infected macrophages (panel A) were incubated in the absence or presence of apigenin (3 μM, 6 μM and 12 μM) for 72 h. ROS generation was measured using the fluorescent dye H_2_DCFDA as described in the Experimental Section. Data are expressed in Fluorescence Intensity Units (FIU). The values shown represent the mean ± standard error of three independent experiments. [* and ** indicate significant differences relative to the control (absence of apigenin) (*p* < 0.05 and *p* < 0.01, respectively)] Panel B: Linear regression analysis was performed using GraphPad Prism 6 (R^2^ = 0.9306). n = 3.

To investigate whether the leishmanicidal effect of apigenin is due to ROS production, ROS levels were measured using the cell-permeable dye H_2_DCFDA [12,13]. Apigenin induced ROS production in *Leishmania*-infected macrophages in a dose-dependent manner (*p <* 0.01) (Fig 2A); however, it did not induce an increase in ROS production in non-infected macrophages, suggesting that such increase is specific to infected cells.

These data suggest that apigenin-induced leishmanicidal activity occurs at least in part through the production of ROS. The linear correlation (R^2^ = 0.9306) observed between the percent inhibition of the infection index and ROS production by apigenin reinforces this hypothesis (Fig 2B). ROS production in a concentration-dependent manner has also been reported for the exposure of *L*. *amazonensis*-infected macrophages to quercetin [12], which induces a severe reduction in the number of parasites. ROS levels were 1.5-fold higher after treatment with 12μM quercetin at 72 h, similar to observations following treatment with apigenin.

**Fig 3:**
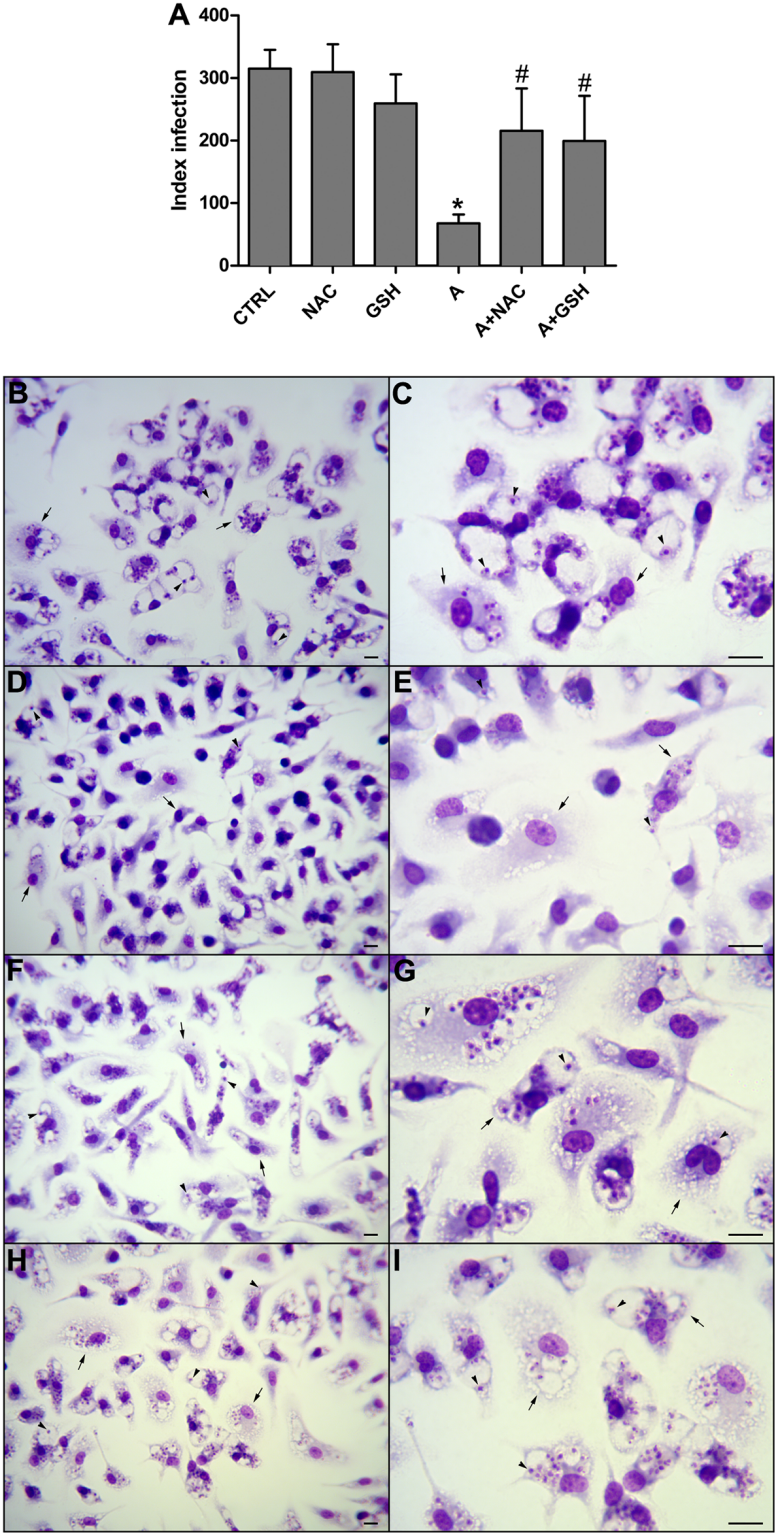
Effect of thiol antioxidants on apigenin-induced leishmanicidal activity. Macrophages were infected with *L*. *amazonensis* promastigotes for 3 h at 37°C as described in the Experimental Section for 72 h in the presence of NAC or GSH in the absence or presence of apigenin. NAC and GSH were solubilized in PBS and added to the culture at a final concentration of 300 μM. Apigenin was solubilized in DMSO (0.2% v/v) and added to the culture at a final concentration of 12 μM. The infection index was determined using light microscopy and counting at least 300 macrophages on each duplicated coverslip. The values shown are the means ± standard errors of three different experiments. In the control (absence of apigenin), the same volume of vehicle (0.2% DMSO) was added to the growth medium. *Leishmania*-infected macrophages were either untreated (Panels B and C) or treated with apigenin (Panels D and E), NAC (Panels F and G) or GSH (Panels H and I). The macrophages were fixed onto glass slides. The slides were stained with the Instant Prov hematological dye system and photographed. The arrows indicate *Leishmania amazonensis*-infected macrophages; intracellular amastigote are indicated by arrowhead. Scale bars correspond to 1 μm. CTRL—control; NAC—*N*-acetyl-L-cysteine; GSH—reduced glutathione. [* indicates significant difference relative to the control group (*p* < 0.05); ^#^ indicates a significant difference relative to the apigenin-treated group (*p* < 0.05)]. n = 3.

In animal cells, reduced glutathione (GSH) is the most abundant non-protein sulfhydryl-containing (thiol) tripeptide. It serves as a cellular defense mechanism against oxidative injury and maintains a reduced cellular environment in many cell types [25]. ROS are often targeted by GSH in both spontaneous and catalytic reactions. *N*-acetyl-L-cysteine (NAC) is a thiol compound that is known to promote GSH synthesis and has been used in conditions characterized by decreased GSH or oxidative stress [26]. To confirm that the inhibitory effects of apigenin are mediated by ROS production, *L*. *amazonensis*-infected macrophages were preincubated with GSH or NAC (300 μM). As demonstrated in Fig 3, GSH and NAC protected *L*. *amazonensis* from apigenin-mediated inhibition (panel A) (*p <* 0.05), corroborating ROS production as a possible mechanism for the induction of *L*. *amazonensis* amastigote death. Treatment with apigenin inhibited the parasite intracellular proliferation without any apparent host cytotoxicity, as evidenced by the intact cell morphology (Fig 3B–3I).

**Fig 4:**
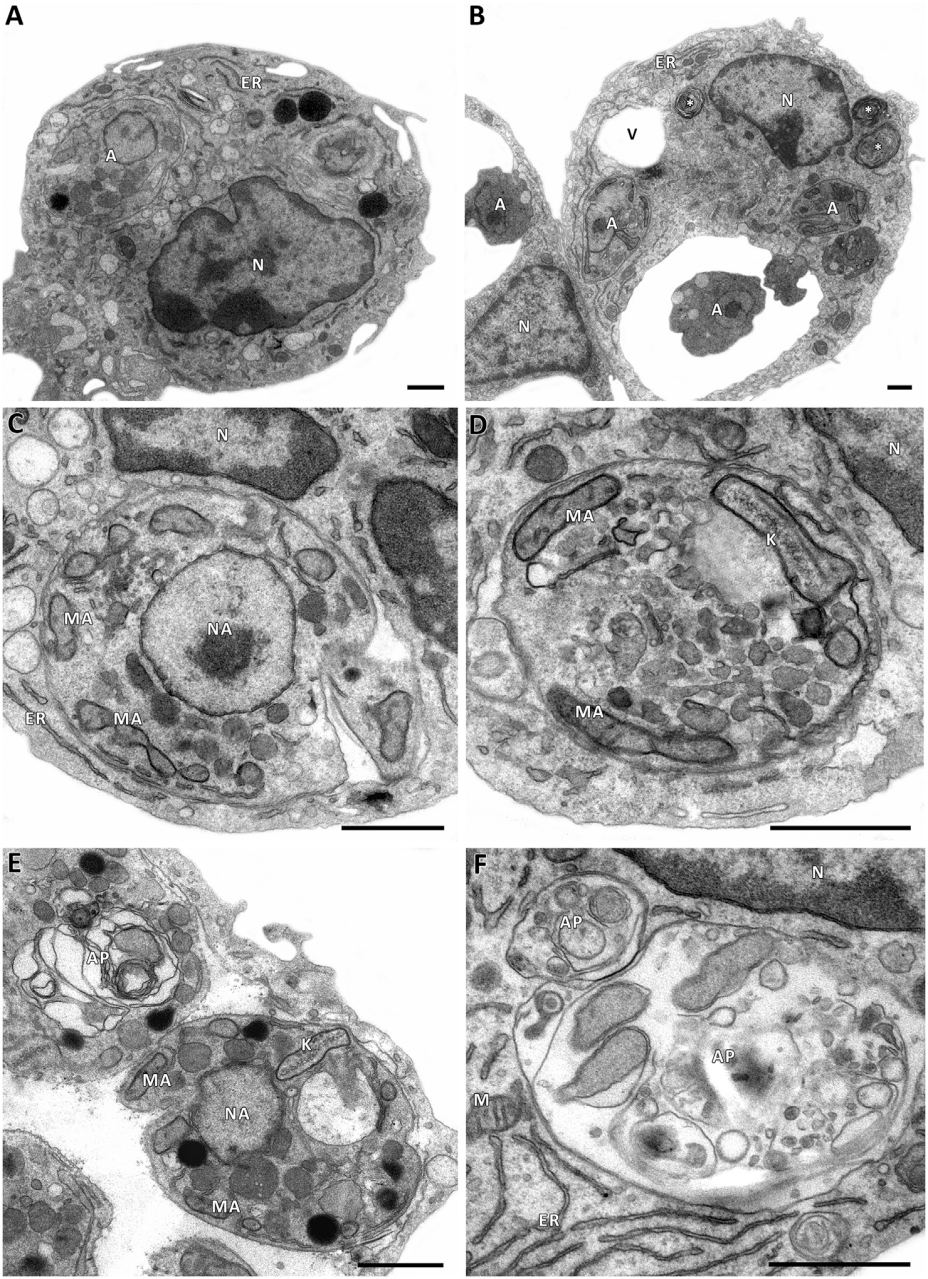
Transmission electron microscopy analysis of untreated *L*. *amazonensis*-infected macrophages. Panels A and B: Infected macrophages demonstrate intact nuclei (N), endoplasmic reticulum (ER), small vacuoles (V), concentric membranous structures (white asterisk) and parasitophorous vacuoles containing amastigotes (A). Panels C and D: Untreated amastigote displaying typical morphology with normal kinetoplast (K), mitochondria (MA), and nucleus (NA). Panel E: Autophagosomes (AP) were observed surrounding the amastigote with intact morphological structures; mitochondria (MA), nucleus (NA) and kinetoplast (K). Panel F: Infected macrophage showing autophagosomes (AP) close to mitochondria (M), the nucleus (N) and the endoplasmic reticulum (ER). Scale bars correspond to 1 μm.

**Fig 5:**
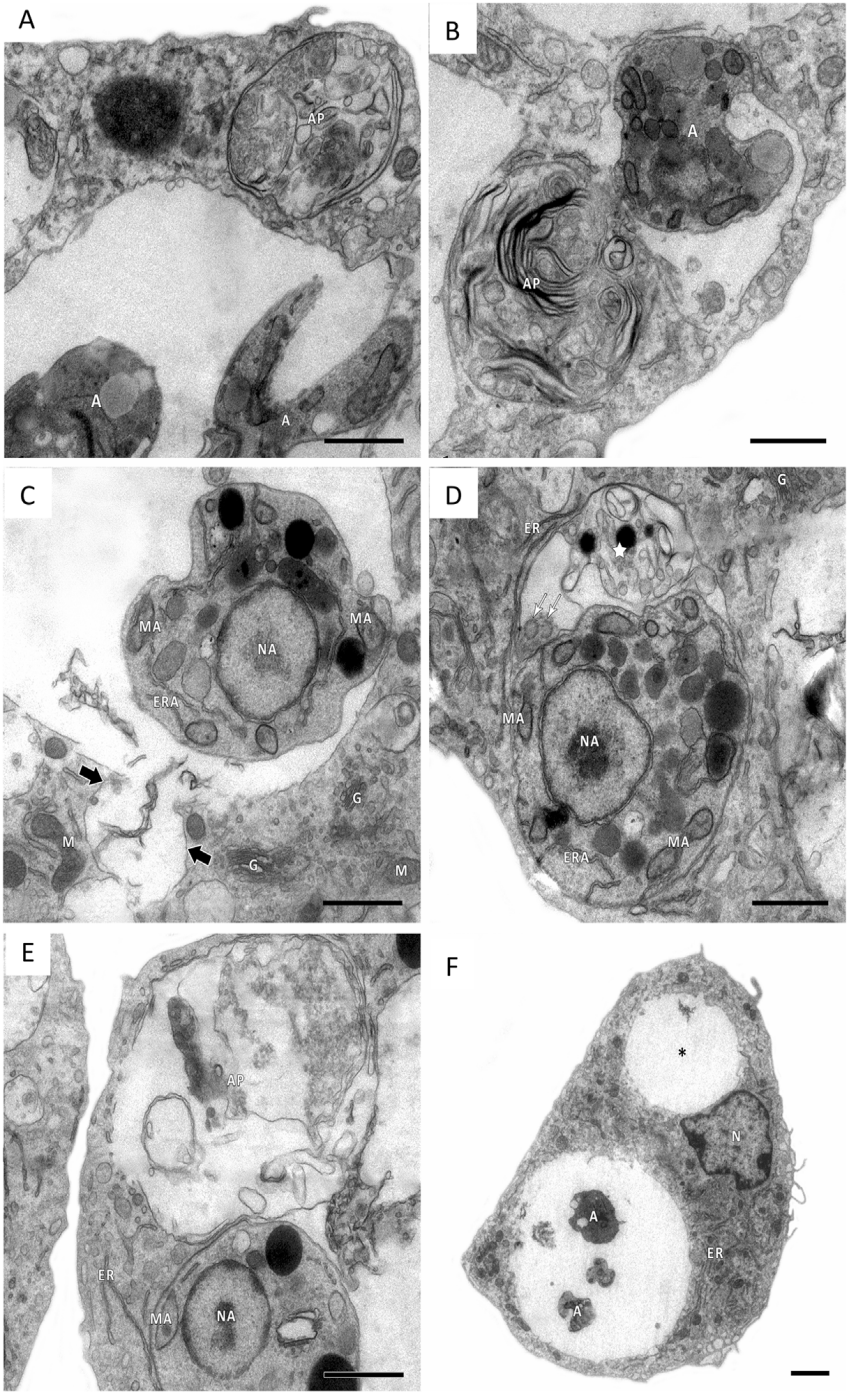
Ultrastructural analysis of *L*. *amazonensis*-infected macrophages treated with apigenin. *Leishmania*-infected macrophages were incubated in the presence of apigenin (12 μM) for 72 h as described in Experimental Section. Panels A and B: Treatment induced a marked increase in the autophagosomes (AP) surrounding the amastigotes (A). Panel C: Fusion between autophagosomes-like compartments and parasitophorous vacuole (black arrow). Panel D: Degraded amastigote (white star) inside the parasitophorous vacuole presenting an axoneme-like structure (white arrow). Panel E: Autophagosomes (AP) proximal to amastigotes (A). Panel F: Infected-macrophage containing a parasitophorous vacuole with amastigotes (A) and an empty parasitophorous vacuole (black star). Morphological structure of macrophage: G—*trans*-Golgi network; M—mitochondria; N—nucleus; ER—endoplasmic reticulum. Morphological structure of amastigote: NA—nucleus; MA—mitochondria; ERA—endoplasmic reticulum. Scale bars correspond to 1 μm.

Autophagy is another mechanism of defense against intracellular pathogens. ROS have been shown to activate autophagy to protect cells from invading pathogens such as *Leishmania* [27]. Transmission electron microscopy analyses of untreated L. amazonensis-infected macrophages are shown in Fig 4. The macrophages displayed typical morphology with a preserved nucleus (N), endoplasmic reticulum (ER) and parasitophorous vacuoles containing amastigotes (A). Concentric membranous structures (white asterisk) and cytosolic vacuolization (V) were also observed (panels A–D). In addition, several autophagosomes (AP) were observed surrounding the parasites (panels E and F). In contrast, when *Leishmania*-infected macrophages were incubated in the presence of apigenin (12 μM) for 72 h, the treatment induced a remarkable increase in the number of double-membrane vesicles and myelin-like membrane inclusions within macrophages, characteristics of autophagic pathway (Fig 5A and 5B); additionally, these structures were found to be co-localized with *L*. *amazonensis* amastigotes (Fig 5; panel B and panel E). Fusion between autophagosomes-like structures and the parasitophorous vacuole was also observed (Fig 5C; black arrow). Treated macrophages displayed both an intact nucleus (N) and endoplasmic reticulum (ER) (Fig 5F).

Increases in pro-oxidant states, promoted by xenobiotic exposure, have been associated with the stimulation of the autophagic pathway, [28] and ROS have been demonstrated as signaling molecules in starvation-induced autophagy [29]. Several sources of ROS exist in phagocytic cells, the most prominent of which is NADPH oxidase (NOX).[30] It has been demonstrated that NADPH oxidase—generated ROS contribute to autophagic induction [27].

Appropriate signaling promotes NOX attachment to the phagosomal membrane and generates superoxide by transferring electrons from cytosolic NADPH to oxygen in the phagosome lumen [31,32]. Increased NOX activation enhances the ability of the infected macrophage to kill *L*. *amazonensis* [33]; conversely, inhibition of NOX activation appears to be a strategy of *L*. *amazonensis* infection [34].

Therefore, it can be postulated that the effects observed following treatment of *L*. *amazonensis*-infected macrophages with apigenin occur through the activation of NOX, generating ROS and leading to an increase in autophagy. This hypothesis is reinforced by the following observations: (a) ROS production occurred only in *L*. *amazonensis*-infected macrophages treated with apigenin, which exhibited a linear correlation between the percent inhibition of the infection index and ROS production; (b) GSH and NAC significantly reduced apigenin-induced intracellular amastigote death; and (c) Apigenin clearly induced a significant increase in autophagosomesco-localized with *L*. *amazonensis* amastigotes in apigenin-treated macrophages without apparent cytotoxicity. Accordingly, it has been demonstrated that in HepG2 human hepatoma cells, activation of NOX leads to ROS generation following treatment with apigenin [16].

The current lack of reasonable therapeutics necessitates the development of novel antileishmanial compounds. A compound is classified as orally effective when it demonstrates good absorption. To determine the possible oral effectiveness of apigenin prior to *in vivo* testing, the ADMET (absorption, distribution, metabolism, excretion and toxicity) properties were evaluated using the admetSAR tool [35] (Table 1). Apigenin presented great probabilities (98.9% and 85.4%) for human intestinal absorption (HIA) and Caco-2 cell permeability, respectively. In terms of metabolism, a series of cytochrome P450 were evaluated. Toxicity was also analyzed, and apigenin demonstrated the absence of mutagenic toxicity and carcinogenic effects. Apigenin is also predicted as a class III risk for acute toxicity (compounds with an LD_50_ greater than 500 mg/kg) [35,36]. Taken together, these data suggest that apigenin is safe and orally absorbed.

**Table 1:** Oral bioavailability, predicted ADMET properties, and molecular properties of Apigenin.

	Result	Probability (%)
	**Absorption**	
BBB	+	63.6
HIA	+	98.9
Caco-2	+	85.4
	**Metabolism**	
CYP450 2C9 substrate	NS	78.13
CYP450 2D6 substrate	NS	91.26
CYP450 3A4 substrate	NS	69.07
CYP450 1A2 inhibitor	I	92.22
CYP450 2C9 inhibitor	I	77.46
CYP450 2D6 inhibitor	NI	92.31
CYP450 2C19 inhibitor	I	70.43
CYP450 3A4 inhibitor	I	95.80
	**Toxicity**	
AMES toxicity	-	89.06
Carcinogens	-	91.81
Acute Oral Toxicity	III	70.12
*n*-ROTB (≤10)	1	
	**Lipinski Molecular Descriptor**	
HBA (≤ 10)	5	
HBD (≤ 5)	3	
clogP (≤5)	2.58	
MW (≤ 500)	270.24	

BBB—blood-brain barrier; HIA—human intestinal absorption; + positive; - negative; I—inhibitor; NI—non-inhibitor; NS—non-substrate; *n*-ROTB—number of rotatable bonds; HBA—number of hydrogen bond acceptors; HBD—number of hydrogen bond donors; clogP—logarithm of compound partition coefficient between *n*-octanol and water; MW—molecular weight. A compound is predicted as a class III risk for acute toxicity when the LD_50_ is greater than 500mg/kg [35,36].

Furthermore, Lipinski’s rule of five was calculated [37]. As observed in Table 1, apigenin has five hydrogen bond acceptors, three hydrogen donors, and a molecular weight of 270.2 and a clogP of 2.58, thus fulfilling the Lipinski rule of five.

**Fig 6:**
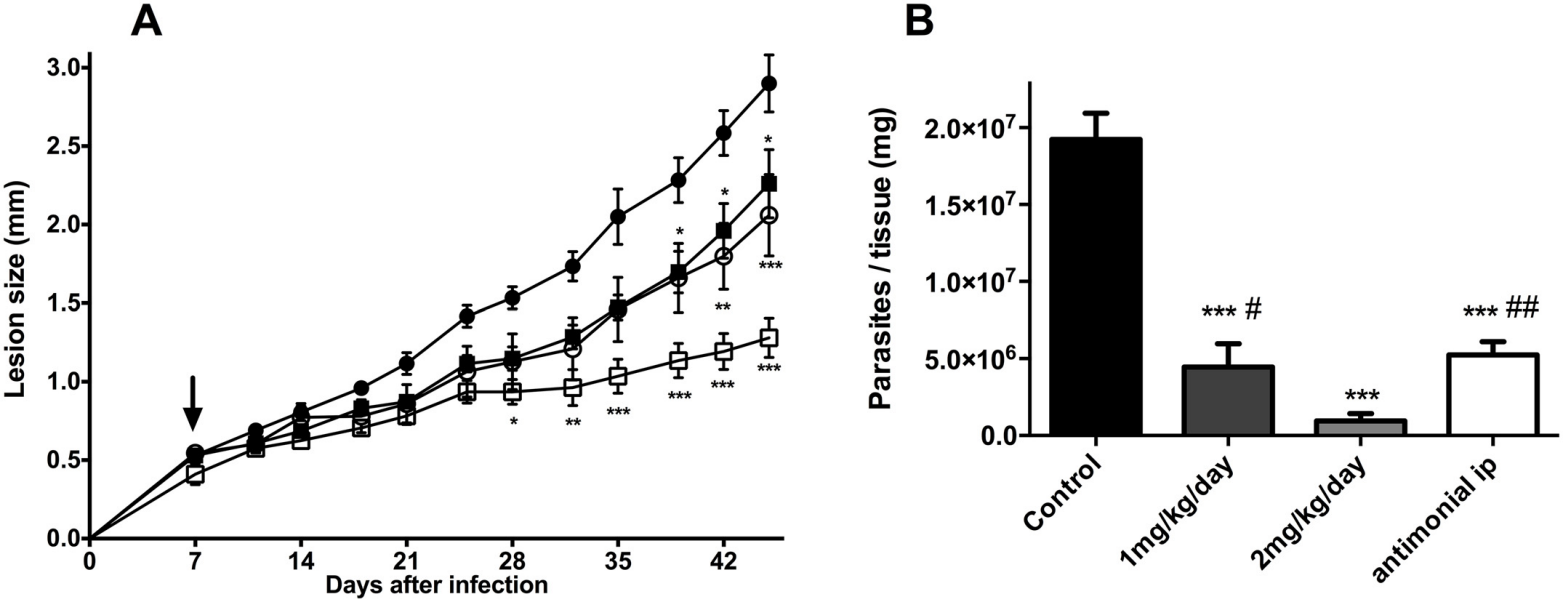
*In vivo* leishmanicidal effect of apigenin using *L*. *amazonensis*-infected BALB/c mice. Mice were infected intradermally with 2 × 10^6^ *L*. *amazonensis* promastigotes in the right ear. Panel A: Lesion development on the animals treated orally with apigenin (1 mg/kg/day; closed square and 2 mg/kg/day; open square), the control group, treated with oral suspension added to DMSO (0.2% v/v) (vehicle of apigenin; closed circle) and meglumine antimoniate (100 mg/kg/day; open circle) once per day, five times per week. Arrow represents the initiation of treatment. Panel B: Parasite burden of the *L*. *amazonensis*-infected BALB/c mice untreated or treated with apigenin (1 mg/kg/day and 2 mg/kg/day) or meglumine antimoniate (100 mg/kg/day). Ear parasite loads were determined via a limiting dilution assay. Data are expressed as means ± standard errors, n = 5 ears. [*, ** and *** indicate significant differences relative to the control group (p < 0.05; p< 0.01 and p < 0.001, respectively) ^#^ indicates a significant difference relative to the apigenin-treated group (2 mg/kg/day) (*p* < 0.05)]. (CTRL = Control; antimonial = meglumine antimoniate; ip = intraperitoneal).

Taking into consideration the above results, the efficacy of apigenin in a murine model of cutaneous leishmaniasis was evaluated using oral administration. Ears of BALB/c mice were intradermally infected with 2x10^6^*L*. *amazonensis* promastigotes, and the mice were subsequently treated orally with apigenin (1 mg/kg/day and 2 mg/kg/day). As shown in Fig 6A, the oral administration of apigenin reduced the lesion (*p <* 0.05).

Interestingly, oral treatment also significantly reduced the parasite burden (*p <* 0.001), with ED_50_ and ED_90_ values of 0.73 and 1.2 mg/kg/day, respectively. This reduction was equal to 73.4% and 94.3% with 1 and 2 mg/kg/day, respectively (Fig 6B). Furthermore, significant differences in lesion size and parasite load were observed between the infected mice treated with apigenin (2 mg/kg/day) and a pentavalent antimonial (meglumine antimoniate).

In addition, no significant differences were observed in serum alanine aminotransferase, aspartate aminotransferase, creatinine, albumin, globulin, total protein, urea or creatine kinase levels between mice treated with apigenin and the control group (S1 Table). All serological toxicology markers were within reference values, suggesting the absence of kidney and liver toxicity. However, further specific toxicity studies, such as genotoxicity, should be performed.

In conclusion, our study suggests that apigenin exhibits leishmanicidal effects against *L*. *amazonensis*-infected macrophages. ROS production, as part of the mechanism of action, could occur through the increase in host autophagy and thereby promoting parasite death. Furthermore, our data suggest that apigenin is effective in the treatment of *L*. *amazonensis*-infected BALB/c mice by oral administration, without noticeable kidney or liver toxicity. The selective *in vitro* activity of apigenin, together with excellent theoretical predictions of oral availability, clear decreases in parasite load and lesion size, and no observed compromises to the overall health of the infected mice encourage us to supports further studies of apigenin as a candidate for the chemotherapeutic treatment of leishmaniasis.

## Supporting Information

S1 Fig Susceptibility of murine macrophages to apigenin. Macrophages were incubated with the indicated concentration of apigenin for 72 h; cell viability was measured using the alamarBlue assay (panel A), and the mitochondrial membrane potential (ΔΨm) was measured using JC-1 (panel B). The values shown represent the means ± standard error of three independent experiments. In the control samples (absence of apigenin), a similar volume of vehicle (0.2% DMSO) was added to the cells. The positive controls for reduction of cellular viability (disrupted cells) and ΔΨm were obtained by adding 0.1% Triton X-100 (T– 0.1% Triton X-100) for the alamarBlue assay and FCCP (200 μM) for the JC-1 assay. [* indicates a significant difference relative to the control group (p < 0.05)].
(TIFF)

S1 Table Toxicity parameters. Serum levels of toxicological markers in the infected BALB/c mice treated were measured as described in the Experimental Section. Reference values were provided by the Program of Technological Development in Tools for Health-PDTIS-FIOCRUZ. Data are expressed as the mean ± standard error, n = 5. ALT—alanine aminotransferase; AST—aspartate aminotransferase; CREA—creatinine; TP—total protein; GLO—globulin; ALB—albumin; CK—creatine kinase.
(DOCX)
