# Supplementary figures and images for: Oral Efficacy of Apigenin against Cutaneous Leishmaniasis: Involvement of Reactive Oxygen Species and Autophagy as a Mechanism of Action
Source: PLoS Negl Trop Dis. 2016 Feb 10;10(2):e0004442. doi: 10.1371/journal.pntd.0004442 (PMC4749305; doi:10.1371/journal.pntd.0004442)

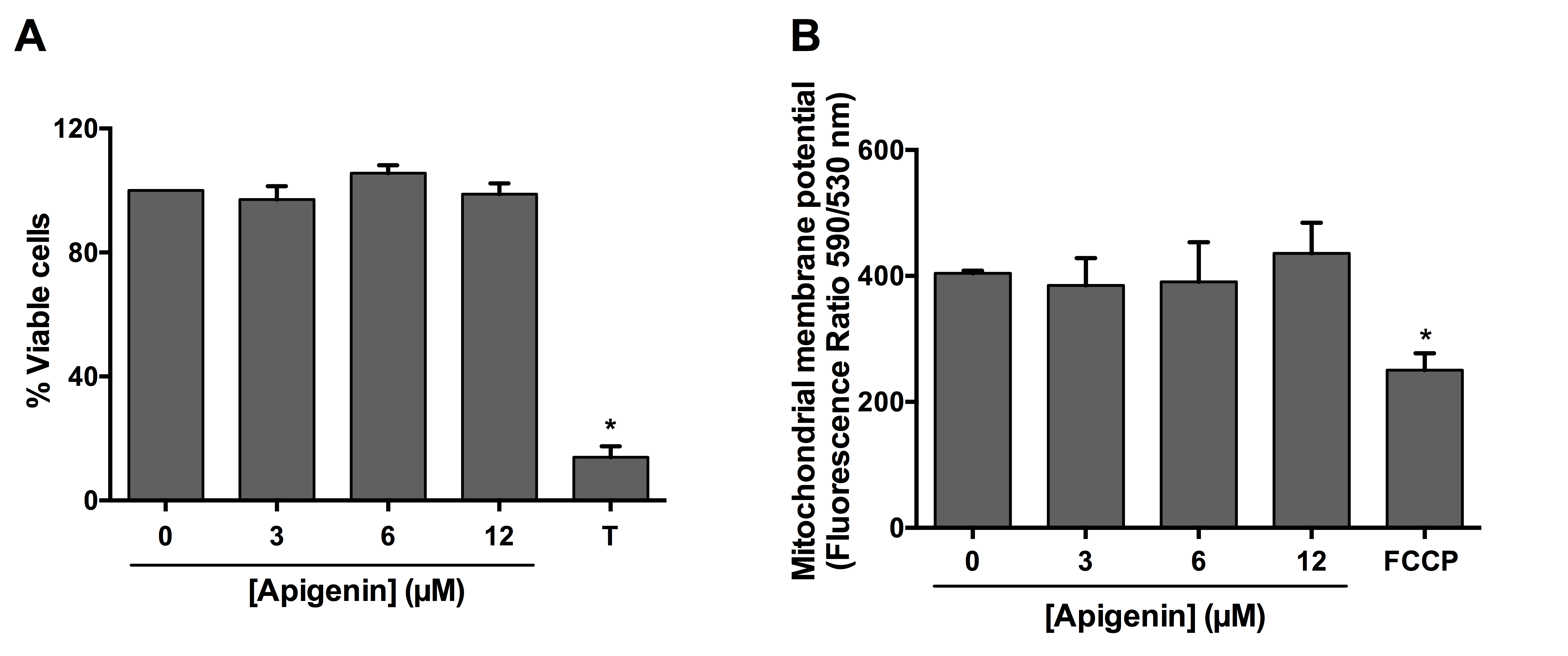

Supplement: S1 Fig — Macrophages were incubated with the indicated concentration of apigenin for 72 h; cell viability was measured using the alamarBlue assay (panel A), and the mitochondrial membrane potential (ΔΨm) was measured using JC-1 (panel B). The values shown represent the means ± standard error of three independent experiments. In the control samples (absence of apigenin), a similar volume of vehicle (0.2% DMSO) was added to the cells. The positive controls for reduction of cellular viability (disrupted cells) and ΔΨm were obtained by adding 0.1% Triton X-100 (T– 0.1% Triton X-100) for the alamarBlue assay and FCCP (200 μM) for the JC-1 assay. [* indicates a significant difference relative to the control group (p < 0.05)]. (TIFF) [file pntd.0004442.s001.tiff]
