# Supplementary material for: Oral Efficacy of Apigenin against Cutaneous Leishmaniasis: Involvement of Reactive Oxygen Species and Autophagy as a Mechanism of Action
Source: PLoS Negl Trop Dis. 2016 Feb 10;10(2):e0004442. doi: 10.1371/journal.pntd.0004442 (PMC4749305; doi:10.1371/journal.pntd.0004442)
Supplement: S1 Table — Serum levels of toxicological markers in the infected BALB/c mice treated were measured as described in the Experimental Section. Reference values were provided by the Program of Technological Development in Tools for Health-PDTIS-FIOCRUZ. Data are expressed as the mean ± standard error, n = 5. ALT—alanine aminotransferase; AST—aspartate aminotransferase; CREA—creatinine; TP—total protein; GLO—globulin; ALB—albumin; CK—creatine kinase. (DOCX) [file pntd.0004442.s002.docx]

|  | **ALT** | **AST** | **CREA** | **Urea** | **TP** | **GLO** | **ALB** | **CK** |
| --- | --- | --- | --- | --- | --- | --- | --- | --- |
| ***Reference values*** | ***28–132 U/L*** | ***59–247 U/L*** | ***0.20–0.80 mg/dL*** | ***18–29 mg/dL*** | ***3.60–6.60 g/dL*** | ***0–6.00 g/dL*** | ***2.50–4.80 g/dL*** | ***68–1070 U/L*** |
| Control | 22.8±0.8 | 69.5±5.8 | 0.215±0.03 | 33.9±1.0 | 4.0±0.2 | 2.6 ± 0.1 | 1.3±0.1 | 608±74.2 |
| Apigenin **1** mg/Kg/dia | 21.6±1.1 | 71±9.8 | 0.222±0.02 | 34.1±1.0 | 4.1±0.3 | 2.7 ± 0.1 | 1.5±0.2 | 546.6±139.8 |
| Apigenin **2** mg/Kg/dia | 21.0±1.8 | 61.8±10.0 | 0.217±0.01 | 37.8±1.1 | 4.0±0.3 | 2.7 ± 0.1 | 1.3±0.2 | 498.8±127.8 |
| pentavalent antimonial | 21.1±1.7 | 62±7.8 | 0.278±0.02 | 36.3±2.0 | 3.9±0.2 | 2.7 ± 0.1 | 1.3±0.2 | 369±102.9 |

**S1 Table**
